# Cadmium exposure and endometrial cancer risk: A large midwestern U.S. population-based case-control study

**DOI:** 10.1371/journal.pone.0179360

**Published:** 2017-07-24

**Authors:** Jane A. McElroy, Robin L. Kruse, James Guthrie, Ronald E. Gangnon, J. David Robertson

**Affiliations:** 1 Department of Family & Community Medicine, School of Medicine, University of Missouri, Columbia, Missouri, United States of America; 2 University of Missouri, Research Reactor Center, Columbia, Missouri, United States of America; 3 Departments of Biostatistics & Medical Informatics and Population Health Sciences, School of Medicine and Public Health, University of Wisconsin-Madison, Madison, Wisconsin, United States of America; 4 Department of Chemistry, University of Missouri, Columbia, Missouri, United States of America; University of Oxford, UNITED KINGDOM

## Abstract

Estrogen-mimicking chemicals, such as cadmium, may be associated with increased susceptibility to hormone-dependent cancers, though supporting data are sparse, particularly for endometrial cancer. The Health and Environmental Exposure Research (HEER) study worked with the Arkansas Central Cancer Registry, Iowa Cancer Registry and Missouri Cancer Registry to obtain names of women diagnosed with endometrial cancer who were willing to be contacted for participation in our case control study. Voter registration lists from Iowa and Missouri were used to randomly select similarly aged women as represented in the case population. Participants were interviewed by telephone to obtain information on known or suspected endometrial risk factors. Urine kits were sent to participants for home collection and returned for analysis. Our case-control study consisted of 631 incident cases of endometrial cancer diagnosed from January 2010 to October 2012 and 879 age-matched population-based controls, ages 18–81 years (mean age 65 years). We quantified cadmium amounts in urine and standardized these values through creatinine adjustment. Using data from all survey completers, we developed a multivariable model for endometrial cancer. Creatinine-adjusted cadmium concentration was added to this model. Odds ratio (OR) and 95% confidence intervals (CIs) for endometrial cancer were calculated. After multivariable adjustment, higher creatinine-adjusted cadmium exposure was associated with a statistically significant increase of endometrial cancer risk (OR: 1.22; 95% CI: 1.03–1.44). Our results provide evidence that cadmium may increase the risk of endometrial cancer, possibly through estrogenic effects.

## Introduction

Cadmium is a toxic, bioaccumulating, non-essential, and highly persistent metal with a variety of adverse health effects. These include renal dysfunction; breast, lung, pancreatic, and endometrial cancer risk; and disturbances in calcium homeostasis [1–5]. Ingesting food containing cadmium (e.g., kidney, liver, crustaceans, cereals) is the primary non-occupational source [6,7]. Smoking is the second major source of cadmium exposure in the general population [7]. Tobacco plants readily take up agricultural sources of cadmium [8]. A doubling of urinary cadmium levels may be found in heavy smokers (>300 pack years (number of smoking years times usual number of cigarettes per day)) compared to non-smokers [9].

Because the estimated biological half-life of cadmium is from 10 to 30 years [10], only a small fraction of inhaled or ingested cadmium is excreted, and the body burden increases over time. Women generally have higher cadmium levels than men [11], and parous women are more likely to have depleted iron stores and therefore absorb more cadmium when compared to nulliparous women [12].

Endometrial cancer, the fourth most common cancer for women, occurs primarily in postmenopausal women. In 2016, approximately 60,050 women are expected to be diagnosed with cancer of the body of the uterus (endometrial cancers and uterine sarcomas), with 10,470 projected deaths [13]. Endometrial cancer is associated with both endogenous and exogenous estrogen exposure [14]. Cadmium mimics estrogen and may increase estrogen-receptor-mediated proliferation—the classical estrogen signaling, thereby potentially contributing to endometrial cancer risk [15,16]. Two nuclear estrogen receptors, (estrogen receptor alpha (ERα) and estrogen receptor beta (ERβ)) are involved in the classical estrogen signaling. The ERα and ERβ directly interact with specific DNA binding sites that regulate gene expression [17,18]. Alternatively, Ali and colleagues demonstrated that cadmium exposure in mice affected the height of the uterine luminal epithelium in a dose-dependent manner. They suggested that the effects of cadmium exposure was through non-classical estrogen receptor signaling [19]. In 2008, Akesson et al reported a significant association between endometrial cancer and cadmium intake based on assignment of cadmium exposure levels from a food frequency questionnaire [1]. However, more recent studies using food frequency questionnaires to estimate cadmium levels have not reported an association [20–22]. Vacchi-Suzzi [23] reported minimal correlation between urinary cadmium levels and estimates of dietary cadmium intake. This finding and inconsistent observational studies using estimated cadmium exposure from self-reported dietary intake has brought the validity of food frequency questionnaires for cadmium estimation into question [24–26].

The Health and Environmental Exposure Research (HEER) study aimed to investigate the association between endometrial cancer risk from cadmium exposure after adjusting for confounders in a population-based case-control study using cases from the Arkansas, Iowa and Missouri state cancer registries and age-matched controls from voter registration lists.

## Materials and methods

### Study population

Incident cases of endometrial carcinoma diagnosed from January 2010 to October 2012 were obtained from three cancer registries: Arkansas Central Cancer Registry, Iowa Cancer Registry and Missouri Cancer Registry. In situ and non-carcinomas, such as Müllerian and mesodermal malignant tumors, were excluded. Case ascertainment also included local or regional stages, excluding metastatic endometrial cancer cases. Cases were contacted by each registry to obtain written permission to pass their names to our study. Of those that agreed, contact information was sent to HEER study staff.

Age-matched controls were randomly selected from the Iowa and Missouri voter registration lists only as the Arkansas voter registration list was not available. The Iowa voter registration list provided date of birth, whereas the Missouri voter registration list provided age. For Missouri controls, the age of the participant was at the last voter registration period and was thus approximately one to three years younger than the current year. We therefore used the listed age plus 2 years for matching. For each case, four to five controls were randomly selected from the voter registration lists with replacements on names for which the telephone number or residential address in Missouri or Iowa was missing. Imperfect age matching occurred due to variable responses by potential controls. The age or year of cancer diagnosis for cases was used as the reference age or year for the matched controls, respectively. Most questions asked for information during the year before diagnosis, i.e. reference age/year minus one.

Introductory letters with a study brochure and consent information were sent to all potential participants. For participants who did not contact the study to decline participation and for whom a valid address was obtained, contact information was sent to the University of South Carolina Call Center for telephone interviews.

### Assessment of cadmium and covariates

All participants were interviewed by telephone by trained interviewers. The 35-minute interview asked about physical activity, reproductive history, alcohol consumption, height and weight, use of oral contraceptives and hormone replacement therapy, personal and family medical history, demographic factors, a limited set of dietary components, and smoking history.

Upon completion of the telephone interview a kit to collect saliva and urine at home and return to the study center was sent to participants. Urine sample containers and urine hats used to collect the sample were exhaustively cleaned using multistep acid leachings before sending to participants [27]. Analyses of acid leaching solutions from kit materials revealed no detectable cadmium. Urine collection containers were used to prepare method blanks. Detailed photo-essay instructions were carefully designed for the urine collection kits to minimize trace element contamination during specimen collection and handling.

Urine samples were immediately refrigerated (4^o^ Celsius) upon receipt. The University of Missouri Research Reactor (MURR) has a laboratory that is specially designed for trace element analysis and is subject to high-efficiency particulate air filtration. The trace-analysis laboratory at MURR has been has been conducting elemental analyses of biological monitors for 35 years [28]. Cadmium was quantified by using inductively coupled plasma-dynamic reaction cell-mass spectrometry (ICP-DRC-MS) following an established protocol from the National Health and Nutritional Examination Study (NHANES) [29], with an additional correction for the effect of strontium on the tin interference. Trace metal analyses were performed from November 2012 to July 2014. A comprehensive quality-control program incorporating numerous methods including method blanks, monitoring multiple cadmium isotopes, internal and external controls, stability samples, replicates, spikes, and routine inclusion of National Institute of Standards and Technology (NIST) standard reference materials (SRM) ensured high-quality data. The instrument detection limit (DL) for cadmium was 0.0017 μg/L, the method detection limit (MDL) was 0.0082 μg/L, and the limit of quantification (3.3 x MDL) was 0.027 μg/L. Cadmium levels in every sample exceeded the MDL, and in no case did the method blank (prepared using urine vials from empty specimen collection kits) exhibit [Cd] > DL. Measurements of NIST SRM 2670a “low” yielded (average ± standard. deviation, % relative standard deviation [RSD]) 0.045 ± 0.006 μg/L, 13%RSD (n = 19); 2670a “high” yielded 4.90 ± 0.16 μg/L, 3.3%RSD (n = 18). Three participant samples were aliquoted and measured repeatedly throughout the study, yielding 0.223 ± 0.016 μg/L, 7.2%RSD (n = 17); 0.184 ± 0.013 μg/L, 7.0%RSD (n = 17); 0.650 ± 0.030 μg/L, 4.7%RSD (n = 17). Urine creatinine level was also measured using a colorimetric assay based on the Jaffé reaction to control for kidney function [30].

This study and all HEER consent documents and procedures were approved by the University of Missouri Health Sciences Institutional Review Board as well as the review boards of the respective state cancer registries. Participants provided written consent to have their names passed to the HEER study. Each cancer registry kept these written consents. Invitation letters were sent to registry-consented participants with an explanation of the study as well as information about the next step—to telephone the participant to conduct the survey. Contact information was also included in the invitation letter for those who did not wish to be called to opt out of being telephoned. Upon telephoning the participants, the study was explained with an opportunity to ask questions to clarify any concerns. Those who agreed to be interviewed were considered to have provided verbally informed consent.

### Statistical analysis

We initially compared creatinine-adjusted cadmium concentration between cases and controls using a t-test. We performed cross-tabulations of survey variables with case-control status to screen for inclusion in a multivariable model. Missing data were relatively rare however we imputed missing values with multiple imputation using the Markov chain Monte Carlo method to impute 20 datasets to be used in the multivariable conditional logistic models. Prior to multiple imputation, we imputed menopausal status based on participant report, smoking status, and use of hormone replacement therapy. A woman was classified as premenopausal if she reported still having periods and was not using hormone replacement therapy. A participant was classified as postmenopausal if she reported an oophorectomy or natural menopause (no menstrual periods for at least 6 months) before the reference date. Women who were taking postmenopausal hormones and still having periods, and women who reported hysterectomy alone were classified as premenopausal if their reference ages were in the first decile of age at natural menopause among the controls (32 years old for current smokers and 36 years old for nonsmokers), and postmenopausal if their reference ages were in the highest decile for age at natural menopause in the control group (56 years old for both current smokers and nonsmokers). For six women in the intermediate age category (second to ninth deciles), menopausal status was considered unknown. Thus, we defined three categories of menopausal status: premenopausal, postmenopausal, and unknown [31].

We developed a multivariable conditional logistic model, stratifying on age at diagnosis. Variables that were selected a priori (race, marital status, body mass index (BMI)), menopause at age 56 or older, menopausal status at diagnosis, smoking history, second hand smoke exposure, age at menarche, number of live births, weight gain, history of weight loss attempt, family history of endometrial cancer in a first degree relative, exposure to unopposed estrogen, oral contraceptive use, history of breast or ovarian cancer, history of uterine fibroids, history of diabetes, sleep habits, irregular work schedule, alcohol use, protein shake and whole milk consumption) were tested for association with case-control status in a multivariable conditional logistic regression. Those with p<0.1 were retained for further analysis. BMI, weight in kilograms divided by squared height in meters, was capped at a maximum value of 70 kg/m^2^ to avoid undue influence of extreme outliers. Variables were selected by both forward stepwise inclusion and backwards elimination, using p = 0.1 for both entry and exit. Both stepwise models retained the same set of variables. Interactions between variables in the model were tested; none were retained.

After the “best” case-control model had been achieved, the base-2 logarithm of creatinine-adjusted cadmium was added to the model to test for its association with case-control status after controlling for other risk factors, using data from women who returned a urine sample. Because the women who opted to provide urine samples differed from those who did not, we developed a multivariable logistic model to adjust for a potential non-response bias in the case-control model [32]. The inverse of the predicted probability of returning a sample was used as a weight in the conditional logistic regression model. The final set of independent variables were race; marital status; BMI; history of weight loss attempt; smoking status; pack-years of cigarette smoking for former and current smokers; history of endometriosis, breast cancer, ovarian cancer or uterine fibroids; family history of endometrial cancer; years of oral contraceptive use; years of unopposed estrogen use; menopause at age 56 years or older; menopausal status at diagnosis; protein shake and whole milk consumption; and non-response bias. We also ran the model with only post-menopausal women and a model that excluded smoking status. SAS for Windows v9.4 (SAS Institute Inc., Cary, NC, USA) was used.

## Results

Of those approached by the cancer registries (n = 2597) for permission to send their names to our study for potential enrollment, 29% of the names (m = 749) were sent to the call center for interviewing (approximately 25% of MO and AR and 44% of IA). Of 711 eligible cases 89% (n = 631) completed an interview. In comparison to those who declined to pass their names to our study from the cancer registries, those who passed their names were more likely to be White (94% versus 90%) or married or living with partner (66% versus 55%) and less likely to be diagnosed with endometrioid carcinoma (International Classification of Diseases for Oncology: 8380) (74% versus 78%). The proportion with tumor grade I or II and age at diagnosis were similar between the two groups.

**Fig 1:**
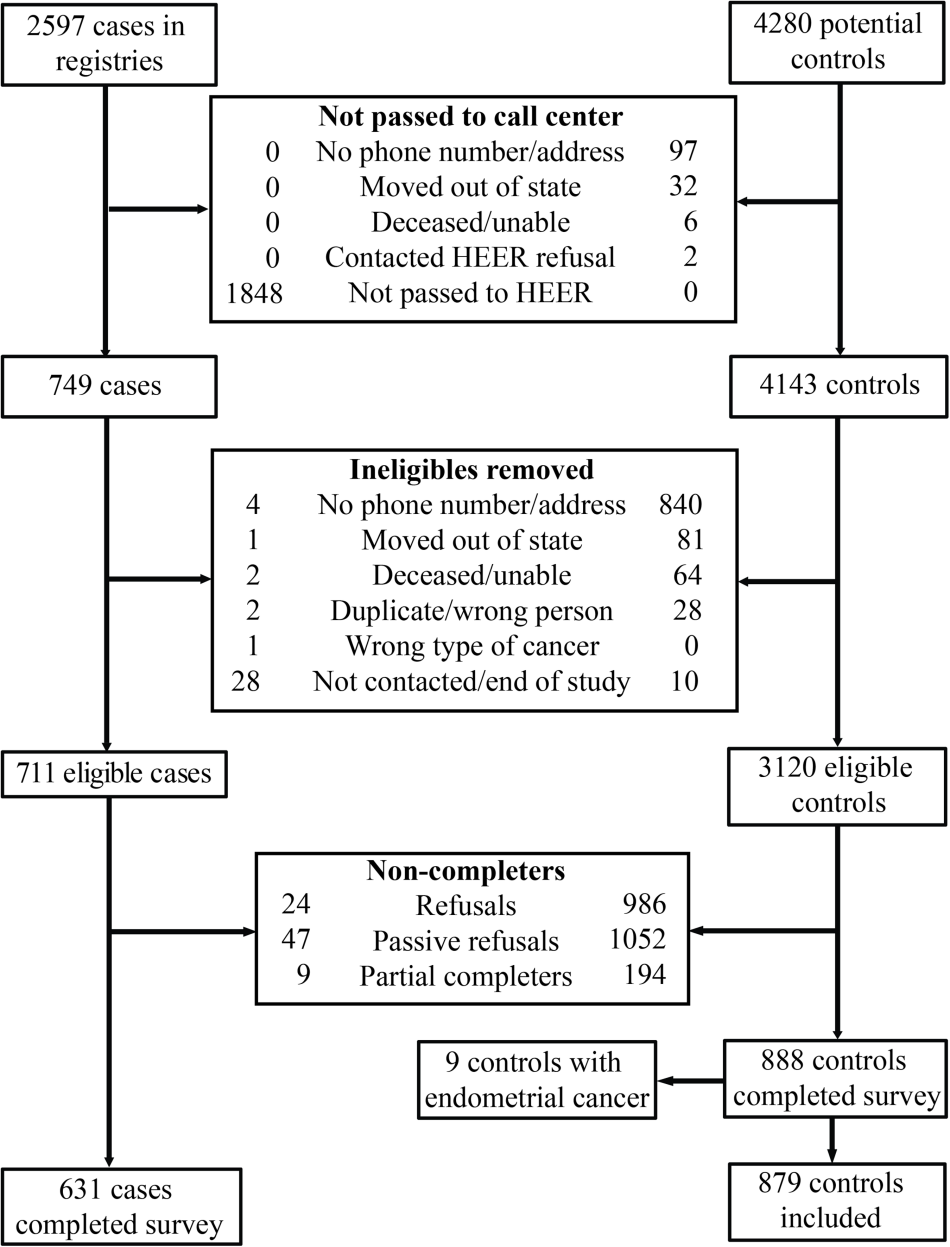
Exclusion and enrollment of participants in the Health and Environmental Exposure Research study.

For the controls, 4280 age-matched names were randomly selected from the voter registration lists. Of the 4280 controls, 3120 were eligible and 888 completed the survey (28% participation proportion). Of the 2597 cases, 749 women with endometrial cancer were passed to the HEER study and 631 completed the survey (24% participation proportion for cases). Among these 1519 participants, 498 cases (79%) and 545 controls (61%) also returned urine specimens. For the current study, we excluded 9 controls with a history of endometrial cancer, leaving 1510 participants (631 cases and 879 controls). See Fig 1 for a summary of participant enrollment.

Controls were slightly older than cases (mean age 63 years versus 60 years, respectively), with less education (high school graduate or less: 43% versus 37%). Both marital status and racial compositions differed between the two groups (Table 1). The cases and controls were similar in income and employment status. Among the cases, 86% had Type I, mostly endometrioid adenocarcinomas, which is driven by hormonal mechanisms, and 14% were Type II, endometrial carcinomas, which often display as serous or clear cell histology [33].

**Table 1:** Characteristics of endometrial cancer cases and population-based controls*.

Characteristic (N missing)	Number (Percent)	Cases (N = 631)	Controls (N = 879)	p-value**
**Demographics**				
Age at diagnosis				< .0001
<40	32 (2.1)	20 (3.2)	12 (1.4)	
40–44	38 (2.5)	24 (3.8)	14 (1.6)	
45–49	54 (3.6)	30 (4.8)	24 (2.7)	
50–54	144 (9.5)	82 (13.0)	62 (7.0)	
55+	1242 (82.2)	475 (75.3)	767 (87.3)	
Mean (SD)	61.7 (9.1)	60.1 (9.5)	62.9 (8.6)	< .0001
Education				
High school or less	607 (40.2)	231 (36.6)	376 (42.8)	.046
1–3 years of college	414 (27.4)	179 (28.4)	235 (26.7)	
College or more	489 (32.4)	221 (35.0)	268 (30.5)	
Employment				.22
Not in labor force	722 (47.8)	290 (46.0)	432 (49.2)	
Working	788 (52.2)	341 (54.0)	447 (50.8)	
Family income, annual (101)				.09
≤ $30,000	417 (29.6)	188 (31.3)	229 (28.3)	
$30,001–$50,000	389 (27.6)	177 (29.4)	212 (26.2)	
$50,001–$100,000	424 (30.1)	160 (26.6)	264 (32.7)	
>$100,000	179 (12.7)	76 (12.6)	103 (12.8)	
Marital status				< .0001
Married/living with partner	1078 (71.4)	417 (66.1)	661 (75.2)	
Divorced, separated, widowed	342 (22.6)	153 (24.2)	189 (21.5)	
Never married	90 (6.0)	61 (9.7)	29 (3.3)	
Race/ethnicity (6)				
Hispanic	11 (0.7)	5 (0.8)	6 (0.7)	.79
Non-Hispanic Black	37 (2.5)	26 (4.1)	11 (1.3)	.0004
Non-Hispanic White	1437 (95.6)	595 (94.6)	842 (96.2)	
Other Non-Hispanic	19 (1.3)	3 (0.5)	16 (1.8)	.024
Work schedule (723)				.32
Regular daytime shift	650 (82.6)	286 (84.1)	364 (81.4)	
Other shift	137 (17.4)	54 (15.9)	83 (18.6)	
**Dietary habits**				
Drink alcoholic beverages, days/week (18)				
0	706 (47.3)	290 (46.5)	416 (47.9)	.07
<1	405 (27.1)	190 (30.4)	215 (24.8)	
1–2.9	183 (12.3)	70 (11.2)	113 (13.0)	
3 or more	198 (13.3)	74 (11.9)	124 (14.3)	
Protein shake consumption (5)				.02
None	1328 (88.2)	542 (86.0)	786 (89.8)	
Less than 2 per week	73 (4.8)	31 (4.9)	42 (4.8)	
2 per week or more	104 (6.9)	57 (9.0)	47 (5.4)	
Whole milk consumption				.038
5 or more times per week	75 (5.0)	40 (6.3)	35 (4.0)	
Less than 5 times per week	1435 (95.0)	591 (94.7)	844 (96.0)	
**Physical activity and weight**				
Body mass index, year before diagnosis (20)				
Mean (SD)	32.0 (8.9)	35.7 (9.9)	29.3 (7.0)	< .0001
MET-hours/week, total (8)				
Mean (SD)	38.2 (58.9)	32.5 (51.5)	42.3 (63.4)	.001
Weight gain since age 25 (23)				
Mean (SD)	20.5 (18.3)	25.0 (20.4)	17.2 (15.9)	< .0001
**Smoking history**				
Smoke cigarettes (3)				.003
Never	946 (62.8)	413 (65.7)	533 (60.7)	
Former	418 (27.7)	175 (27.8)	243 (27.7)	
Current	143 (9.5)	41 (6.5)	102 (11.6)	
Smoking, total pack-years (6)				
Mean for ever-smokers (SD)	9.0 (18.8)	7.6 (13.5)	10.9 (16.7)	.0002
**Medical history**				
Breast cancer (3)				.0002
Yes	75 (5.0)	16 (2.5)	59 (6.7)	
No	1432 (95.0)	614 (97.5)	818 (93.3)	
Diabetes (4)				
Yes	291 (19.3)	155 (24.7)	136 (15.5)	< .0001
No	1215 (80.7)	476 (75.3)	742 (84.5)	
Endometrial cancer in first degree relative (67)				.0001
Yes	48 (3.3)	33 (5.5)	15 (1.8)	
No	1395 (96.7)	570 (94.5)	825 (98.2)	
Endometriosis (31)				.07
Yes	250 (16.9)	116 (19.0)	134 (15.4)	
No	1229 (83.1)	494 (81.0)	735 (84.6)	
Hypertension (8)				.008
Yes	799 (53.2)	359 (57.3)	440 (50.3)	
No	703 (46.8)	268 (42.7)	435 (49.7)	
Ovarian cancer (10)				< .0001
Yes	46 (3.1)	35 (5.6)	11 (1.3)	
No	1454 (96.9)	590 (94.4)	864 (98.7)	
**Medications and treatments**				
Birth control, years used (7)***				
Mean (SD)	5.8 (7.2)	4.8 (6.0)	6.5 (7.9)	< .0001
Hormone replacement therapy, years used (22)				
Mean (SD)	3.2 (7.1)	2.0 (5.6)	4.1 (7.9)	< .0001
**Reproductive history**				
Age of menarche 11 or earlier				.004
Yes	330 (21.8)	161 (25.5)	169 (19.2)	
No	1180 (78.2)	470 (74.5)	710 (80.8)	
Menopause status, year before diagnosis (6)				< .0001
Premenopausal	220 (14.6)	149 (23.6)	71 (8.1)	
Postmenopausal	1284 (85.0)	481 (76.2)	803 (91.4)	

*9 controls who reported a diagnosis of endometrial cancer were excluded. Variables refer to the year prior to diagnosis of endometrial cancer unless otherwise specified.

**P-value for chi-square analysis except for means, which were compared with a t-test. Fisher’s Exact test was used for race/ethnicity where there were low numbers of participants in one category.

***Birth control refers to methods that release hormones, such as birth control pills, injections, patches, progestogen implants (Norplant), progestin-releasing intrauterine devices, or vaginal rings.

### Characteristics of refusal at enrollment

Of those who actively declined to participate, 17% (9 cases and 194 controls) agreed to answer a few demographic questions. Compared to participants who completed the interview, these helpful refusals were more likely to be high school graduate or less (40% versus 54%) and be married or living with partner (71% versus 82%). The educational attainment of spouse or partner, percent of Hispanic ethnicity, race, income and sexual orientation were similar between these two groups.

### Reliability substudy

Of the 165 respondents contacted for a second interview, 2 individuals refused to be interviewed, 29 could not be reached, and 134 were re-interviewed (81%; 69 cases and 65 controls). Mean time between interviews was 13.9 months (range 3.1–20.8 months). Time between interviews was not different between cases and controls.

Participation in moderate and vigorous physical activity showed good concordance between interviews (93% and 82% respectively); the kappa for moderate physical activity was 0.54 (lower confidence limit (LCL) 0.27) and vigorous physical activity was 0.64 (LCL 0.51). History of ever using mineral supplements showed good concordance (84.2%) with a low kappa (0.27, LCL 0.05). The kappa for BMI category was .84 (LCL .75) with 89.6% concordance. There was high concordance for ever being diagnosed with polycystic ovarian syndrome (97%); the kappa was 0.48 (LCL 0.05). Ever being diagnosed with diabetes showed high concordance (95.5%); the kappa was 0.85 (LCL .74). Having a biological family member that was ever diagnosed with endometrial cancer had high concordance between interviews (88.1). Correlation between the repeated measures of weight, height, BMI, and age of diabetes diagnosis was high (0.98, 0.97, 0.95, and 0.92, respectively).

### Cadmium analysis

Creatinine-adjusted cadmium levels ranged from to 0.005 to 0.417 (mean 0.037) μg/g in case participants and from 0.006 to 0.649 (mean 0.041) μg/g in control subjects. A t-test of the creatinine-adjusted cadmium concentration between cases and controls was not statistically significant (p = .101).

After multivariable adjustment, a doubling of urine cadmium increased the endometrial cancer risk by 22% (Table 2; odds ratio (OR) = 1.22, 95% confidence interval (CI): 1.03–1.44; p-value = .02) No substantive changes were observed in any parameter estimates when only post-menopausal women were included (S1 Table). A similarly elevated endometrial risk for cadmium exposure was also observed among those diagnosed with Type I endometrial carcinoma (n = 550) compared to controls (OR: 1.21 95% CI 1.03–1.47). Women diagnosed with endometrial carcinoma who were also 50 pounds over ideal weight had an increased endometrial cancer risk for cadmium exposure (S2 Table). When we re-ran the model and did not include current smoking, cadmium concentration remained statistically significant (OR: 1.19, 95% CI: 1.01–1.41) (S3 Table). A similarly elevated endometrial risk for cadmium exposure was also observed when we re-ran the model and include two additional established risk factors, age of menarche and number of live births, (S4 Table). We also re-ran the model and included creatinine concentration as a separate covariant and base-2 logarithm of unadjusted cadmium concentration. Cadmium concentration remained statistically significant (S5 Table).

**Table 2:** Multivariable conditional logistic regression of risk factors for endometrial cancer.

	Base model with all participants		Model with adjusted Cd* and inverse probability weights	
Characteristic	Odds ratio (95% CI)	P-value	Odds ratio (95% CI)	P-value
Non-Hispanic African-American race	3.07 (1.33, 7.10)	.0085	4.91 (1.88, 12.82)	.0012
Marital status (reference never married)				
Married, living with partner	0.47 (0.27, 0.82)	.0077	0.36 (0.17, 0.77)	.0088
Divorced, separated, widowed	0.58 (0.32, 1.06)	.0791	0.44 (0.20, 1.00)	.0497
Body Mass Index at diagnosis^†^	1.08 (1.06, 1.10)	< .0001	1.09 (1.06, 1.11)	< .0001
History of trying to lose weight	1.75 (1.18, 2.60)	.0058	1.59 (0.99, 2.57)	.0573
Current smoker	0.52 (0.32, 0.85)	.0086	0.52 (0.28, 0.96)	.0381
Cigarette smoking (10 pack-years)	0.94 (0.87, 1.01)	.0864	0.88 (0.80, 0.97)	.0086
History of endometriosis	1.64 (1.17, 2.29)	.0037	1.66 (1.10, 2.50)	.0151
History of breast cancer	0.47 (0.25, 0.88)	.0182	0.39 (0.16, 0.93)	.0337
History of ovarian cancer	4.27 (1.89, 9.69)	.0005	9.99 (2.67, 37.38)	.0006
History of uterine fibroids	0.77 (0.57, 1.03)	.0797	0.71 (0.50, 1.00)	.0508
Endometrial cancer in first degree relative	2.70 (1.32, 5.52)	.0065	3.31 (1.37, 8.01)	.0079
Oral contraceptive use (5 years)	0.86 (0.79, 0.95)	.0014	0.88 (0.79, 0.97)	.0138
Unopposed estrogen use (5 years)	0.64 (0.53, 0.77)	< .0001	0.67 (0.53, 0.84)	.0006
Menopause at age 56 or later	1.90 (1.33, 2.70)	.0004	1.70 (1.13, 2.56)	.0113
Post-menopausal at diagnosis	0.43 (0.26, 0.70)	.0009	0.33 (0.21, 0.52)	< .0001
Protein shake consumption, days/week	1.12 (1.01, 1.24)	.0276	1.20 (1.04, 1.39)	.0133
Whole milk consumption, ≥ 5 days/week	1.96 (1.13, 3.42)	.0173	2.57 (1.28, 5.15)	.0078
Base-2 logarithm of adjusted cadmium concentration			1.22 (1.03, 1.44)	.0212

CI = confidence interval

*Cadmium concentration adjusted by urine concentration of creatinine

^†^Body mass index is weight in kilograms divided by (height in meters)^2^

## Discussion

In this population-based case-control study of Midwestern U.S. women, we found a statistically significant positive association between urine cadmium levels and endometrial cancer risk. Specifically, a 22% increased risk of endometrial cancer was associated with doubling cadmium exposure. Our confidence in the findings is strengthened by our large number of cases, evaluation of those who declined to participate at various points in our study, use of population-based controls that were age-matched to cases, inclusion of a reliability study, the use of urine as the biomarker to ascertain lifetime cadmium exposure, additional analyses to account for potential bias, and using an established protocol for urine analysis with an additional correction factor.

To our knowledge, this is the first published report on cadmium exposure and endometrial cancer risk using urine as a biomarker for cadmium measurement. Four other cohort studies conducted in Japan, Denmark, Sweden, and the United States have reported on this association; all have estimated cadmium exposure using food frequency questionnaires with mixed results. Eriksen et al reported a null finding for cadmium exposure and risk of endometrial cancer (192 endometrial cancer cases over 13 year period) using a Danish population-based prospective study as did Sawada in the Japan Public Health Center-based Prospective Study (75 endometrial cancer cases over 9 year period) [20,34]. The Women’s Health Initiative has also reported a null finding on cadmium exposure and endometrial cancer (1198 endometrial cancer cases over 10 year period) [21]. In contrast, Akesson et al reported an increased endometrial cancer risk (relative risk [RR]: 1.39; 95% CI, 1.04–1.86) among the Swedish Mammography Cohort comprised of postmenopausal women (378 endometrial cancer cases over 16 year period) [1]. In a meta-analysis of dietary cadmium intake and cancer risk, Cho reported an increase cancer risk among studies with Western populations (RR: 1.15; 95% CI 1.08–1.23) particularly for hormone-related cancers (prostate, breast and endometrial) though only two of the aforementioned four cohort studies were included in Cho’s meta-analysis [22]. One recent cancer mortality study using NHANES III data (1988–1994) and creatinine adjusted urinary cadmium found a suggestion of an increased risk for uterine cancer among those with highest level of urinary Cd (n = 7 deaths, mean follow-up 14 years; adjusted hazard ratio (aHR): 1.03; 95% CI 0.23–4.62 and aHR per 2-fold urinary Cd: 1.63; 95% CI 1.06–2.51) [35].

After humans ingest or inhale cadmium, the body excretes only a very small fraction and efficiently retains the rest [7]. Among the potential matrices used to measure cadmium (blood, nail, hair, urine), urine specimens more closely reflect lifetime cadmium exposure than the other matrices [11]. Although not without criticism [36], using creatinine-adjusted values in the field of toxicology for spot urine samples, such as in this study, is common and several papers support this analytic technique [37]. For example, the Jaffé reaction may cause overestimation of creatinine, as mentioned in National Health and Nutrition Examination material [38]. Barr et al suggest using urinary creatinine as an independent variable which allows for urinary dilution and demographic difference adjustment [39]. When we included the base-2 logarithm of unadjusted cadmium concentration and creatinine concentration in our model, results were essentially unchanged from those reported in Table 2. Our unadjusted geometric mean of cadmium was slightly higher (0.32, 95% CI: 0.31–0.34 μg/l) than those reported from 1999–2010 NHANES for women age 20–85 years (0.25, 95% CI: 0.24–0.26 μg/l), though this may reflect a different age structure between these two samples. As noted by Adams and Newcomb, urinary cadmium values for those aged 60–69 years was 2.7 fold greater compared to those 20–29 years old [40].

Cadmium levels are related to level of smoking. Heavy smokers may have twice as much cadmium, and moderate smokers’ cadmium burden may increase by approximately sixty percent compared to non-smokers [9]. Former smokers have a cadmium body burden that is intermediate [24,41]. Smoking has been shown to decrease the risk of endometrial cancer, likely through endometrial atrophy [42]. However, Brinton and others suggest that smoking in conjunction with use of exogenous estrogens significantly multiplies the risk of developing endometrial cancer, especially in thin women [43]. Unfortunately we did not have a sufficient number of thin smokers to confirm this finding and only 6.5% of cases were smokers.

With our extensive survey we were able to consider numerous variables that are known or suspected to increase the risk of endometrial cancer in the logistic regression models. Well documented endometrial cancer risk factors include late menopause, early menarche, nulliparity, and obesity [14]. Among these risk factors, obesity is strongly associated with an increased endometrial cancer risk [43]. Davies et al suggests this is especially true for those who are 50 pounds or heavier than their ideal weight [44]. Additional risk factors that may be associated with endometrial cancer risk include hypertension [45], family history of cancers (breast, endometrial, ovarian, and/or Lynch’s syndrome) [46], history of endometriosis [47], sleep [48], irregular work schedule [49], uterine fibroids [50], alcohol consumption [51], and dietary choices [52], such as milk consumption [53]. We explored the risk of endometrial cancer and consumption of protein shakes. Besides the typical consumption of milk as part of the protein shake, undeclared anabolic androgenic steroids are found in up to 15% of commercially available non-hormonal supplements (i.e., protein drinks) [54]. Though no data are available on this consumption and endometrial cancer, one risk factor of PCOS relates to dysfunction of androgen receptors leading to hyperandrogenism and an increased risk of endometrial cancer [55,56]. Protective factors may be physical activity [57] and oral contraceptive use [58]. Among these risk factors, we found obesity, late age of menopause, selected dietary choices (milk and protein shakes), history of ovarian cancer or endometriosis, and family history of endometrial cancer as characteristics that increased the risk of endometrial cancer. In our analysis, years of oral contraceptive use, years of unopposed estrogen use, history of breast cancer, smoking and being married were protective factors.

Several limitations should be considered in evaluating our results. One limitation was the low participation proportion of women diagnosed with endometrial cancer. Obtaining consent was a two-stage process. Each cancer registry approached eligible cancer cases and required written consent to pass their names to the HEER study. The second stage involved verbal consent upon contact by telephone by the HEER study team. Only 25%-44% of the women agreed to let their respective cancer registry pass their names onto HEER for enrollment. The second stage participation proportion was 89% of the eligible cases; another 1% who declined to participate answered a few questions to obtain a few details. Those not consenting at stage one were more likely to be married or living with partner while those opting to not enroll at stage two were less likely to be married or living with partner. Differences were also observed in racial composition. Those not consenting to pass their names were more likely to be Black, but no difference was observed in declining to enrollment by race. Proportion of tumor grade I and II and age of diagnoses was similar at both stage one and two among the two groups—those consenting and those declining to participate. Among the controls, our participation proportion was 28%. Although we assessed some characteristics between the two groups at the time of interview (n = 203) in our refusal sub-study, we cannot rule out the possibility of selection bias.

As with any case-control study, the risk of recall bias is also a limitation. All data except information about urinary cadmium measurement and tumor characteristics for cases ere self-reported. However, of the few questions we re-asked of participants, the concordance between the two time periods was quite good. Nevertheless, we cannot eliminate the possibility of recall bias.

In conclusion, our results provide evidence that cadmium may increase the risk of endometrial cancer, possibly through estrogenic effects. Further studies that employ urinary cadmium as the biomarker are necessary given the weak association with estimated cadmium from dietary sources. A comprehensive list of suspected and known risk factors should also be collected to fully adjust the regression models.

## Supporting information

S1 Table Multivariable conditional logistic regression of risk factors for endometrial cancer, excluding premenopausal women from the analysis. (DOCX)

S2 Table Multivariable conditional logistic regression of risk factors for endometrial cancer, including weight at least 50 pounds above ideal weight in the model. (DOCX)

S3 Table Multivariable conditional logistic regression of risk factors for endometrial cancer, excluding current smoking status from model. (DOCX)

S4 Table Multivariable conditional logistic regression of risk factors for endometrial cancer, including age at menarche and number of live births. (DOCX)

S5 Table Multivariable conditional logistic regression of risk factors for endometrial cancer, including unadjusted cadmium concentration and creatinine concentration. (DOCX)
