# Supplementary material for: Cadmium exposure and endometrial cancer risk: A large midwestern U.S. population-based case-control study
Source: PLoS One. 2017 Jul 24;12(7):e0179360. doi: 10.1371/journal.pone.0179360 (PMC5524364; doi:10.1371/journal.pone.0179360)
Supplement: S1 Table — (DOCX) [file pone.0179360.s001.docx]

| S1 Table. Multivariable conditional logistic regression of risk factors for endometrial cancer, excluding premenopausal women from the analysis. | | | |
| --- | --- | --- | --- |
| Characteristic | Parameter estimate | Odds ratio (95% CI) | P-value |
| Non-Hispanic African-American race | 1.3575 | 3.89 (1.56, 9.70) | 0.0036 |
| Marital status (reference never married) |  |  |  |
| Married, living with partner | -0.9324 | 0.39 (0.16, 0.96) | 0.0402 |
| Divorced, separated, widowed | -0.7219 | 0.49 (0.19, 1.23) | 0.1289 |
| Body mass index at diagnosis (5kg.m^2^)^a^ | 0.0828 | 1.51 (1.36, 1.69) | <0.0001 |
| History of trying to lose weight | 0.4842 | 1.62 (0.97, 2.70) | 0.0632 |
| Current smoker | -0.7319 | 0.48 (0.22, 1.05) | 0.0647 |
| Cigarette smoking (10 pack-years) | -0.0169 | 0.84 (0.76, 0.94) | 0.0013 |
| History of endometriosis | 0.4569 | 1.58 (1.03, 2.43) | 0.0377 |
| History of breast cancer | -1.1613 | 0.31 (0.12, 0.82) | 0.0177 |
| History of ovarian cancer | 2.8546 | 17.4 (4.12, 73.2) | 0.0001 |
| History of uterine fibroids | -0.3500 | 0.70 (0.49, 1.02) | 0.062 |
| Endometrial cancer in first degree relative | 1.1589 | 3.19 (1.29, 7.88) | 0.0122 |
| Oral contraceptive use (5 years) | -0.0197 | 0.91 (0.81, 1.01) | 0.0807 |
| Unopposed estrogen use (5 years) | -0.0821 | 0.66 (0.52, 0.84) | 0.0009 |
| Menopause at age 56 or later | 0.1539 | 1.17 (1.00, 1.36) | 0.0463 |
| Whole milk consumption, ≥ 5 days/week | 0.9242 | 2.52 (1.26, 5.05) | 0.0091 |
| Base-2 logarithm of adjusted cadmium concentration (ng/g)^b^ | 0.2222 | 1.25 (1.04, 1.50) | 0.0159 |
| CI = confidence interval  ^a^Body mass index is weight in kilograms divided by (height in meters)^2^  ^b^Adjusted by urine concentration of creatinine (mg/dL) | | | |
