# Supplementary material for: Cadmium exposure and endometrial cancer risk: A large midwestern U.S. population-based case-control study
Source: PLoS One. 2017 Jul 24;12(7):e0179360. doi: 10.1371/journal.pone.0179360 (PMC5524364; doi:10.1371/journal.pone.0179360)
Supplement: S2 Table — (DOCX) [file pone.0179360.s002.docx]

| S2 Table. Multivariable conditional logistic regression of risk factors for endometrial cancer, including weight at least 50 pounds above ideal weight in the model. | | | |
| --- | --- | --- | --- |
| Characteristic | Parameter estimate | Odds ratio (95% CI) | P-value |
| Non-Hispanic African-American race | 1.6203 | 5.05 (1.90, 13.4) | 0.0012 |
| Marital status (reference never married) | -0.9766 | 0.38 (0.18, 0.80) | 0.012 |
| Married, living with partner | -0.7803 | 0.46 (0.20, 1.03) | 0.058 |
| Divorced, separated, widowed | 0.0522 | 1.30 (1.13, 1.50) | 0.0003 |
| Body mass index at diagnosis (5kg.m^2^)^a^ | 0.4972 | 1.64 (1.01, 2.67) | 0.044 |
| History of trying to lose weight | -0.6927 | 0.50 (0.27, 0.93) | 0.027 |
| Cigarette smoking (10 pack-years) | -0.0141 | 0.87 (0.79, 0.95) | 0.0034 |
| History of endometriosis | 0.4794 | 1.62 (1.07, 2.44) | 0.023 |
| History of breast cancer | -0.9703 | 0.38 (0.16, 0.91) | 0.031 |
| History of ovarian cancer | 2.3121 | 10.1 (2.66, 38.3) | 0.0007 |
| History of uterine fibroids | -0.3348 | 0.72 (0.50, 1.01) | 0.060 |
| Endometrial cancer in first degree relative | 1.2600 | 3.53 (1.46, 8.54) | 0.0052 |
| Oral contraceptive use (5 years) | -0.0266 | 0.88 (0.79, 0.97) | 0.014 |
| Unopposed estrogen use (5 years) | -0.0793 | 0.67 (0.53, 0.85) | 0.0008 |
| Menopause at age 56 or later | 0.5568 | 1.75 (1.16, 2.64) | 0.0082 |
| Post-menopausal at diagnosis | -1.0893 | 0.34 (0.21, 0.53) | <.0001 |
| Protein shake consumption, days/week | 0.1824 | 1.20 (1.04, 1.39) | 0.013 |
| Whole milk consumption, ≥ 5 days/week | 0.9489 | 2.58 (1.30, 5.14) | 0.0068 |
| Base-2 logarithm of adjusted cadmium concentration (ng/g)^b^ | 0.2219 | 1.25 (1.06, 1.48) | 0.0094 |
| At least 50 pounds above ideal weight^c^ | 0.6709 | 1.96 (1.24, 3.09) | 0.004 |
| CI = confidence interval  ^a^Body mass index is weight in kilograms divided by (height in meters)^2^  ^b^Adjusted by urine concentration of creatinine (mg/dL)  ^c^Among individuals ≥25, an ideal weight is defined as the weight required for a BMI of 24.9, considering the participant’s height. | | | |
