# Supplementary material for: Cadmium exposure and endometrial cancer risk: A large midwestern U.S. population-based case-control study
Source: PLoS One. 2017 Jul 24;12(7):e0179360. doi: 10.1371/journal.pone.0179360 (PMC5524364; doi:10.1371/journal.pone.0179360)
Supplement: S3 Table — (DOCX) [file pone.0179360.s003.docx]

| S3 Table. Multivariable conditional logistic regression of risk factors for endometrial cancer, excluding current smoking status from model. | | | |
| --- | --- | --- | --- |
| Characteristic | Parameter estimate | Odds ratio (95% CI) | P-value |
| Non-Hispanic African-American race | 1.5754 | 4.83 (1.87, 12.52) | 0.0012 |
| Marital status (reference never married) |  |  |  |
| Married, living with partner | -1.0361 | 0.35 (0.17, 0.75) | 0.0065 |
| Divorced, separated, widowed | -0.8275 | 0.44 (0.20, 0.97) | 0.0415 |
| Body mass index at diagnosis (5kg.m^2^)^a^ | 0.0830 | 1.51 (1.37, 1.67) | <.0001 |
| History of trying to lose weight | 0.4796 | 1.62 (1.00, 2.61) | 0.0499 |
| Cigarette smoking (10 pack-years) | -0.0166 | 0.85 (0.77, 0.93) | 0.0003 |
| History of endometriosis | 0.5241 | 1.69 (1.12, 2.54) | 0.0115 |
| History of breast cancer | -0.9421 | 0.39 (0.17, 0.92) | 0.031 |
| History of ovarian cancer | 2.2745 | 9.72 (2.75, 34.4) | 0.0004 |
| History of uterine fibroids | -0.3582 | 0.70 (0.50, 0.99) | 0.041 |
| Endometrial cancer in first degree relative | 1.2292 | 3.42 (1.39, 8.41) | 0.0074 |
| Oral contraceptive use (5 years) | -0.0237 | 0.89 (0.80, 0.98) | 0.0238 |
| Unopposed estrogen use (5 years) | -0.0802 | 0.67 (0.53, 0.84) | 0.0007 |
| Menopause at age 56 or later | 0.5428 | 1.72 (1.15, 2.58) | 0.0086 |
| Post-menopausal at diagnosis | -1.0324 | 0.36 (0.23, 0.56) | <.0001 |
| Protein shake consumption, days/week | 0.1789 | 1.20 (1.04, 1.38) | 0.0152 |
| Whole milk consumption, ≥ 5 days/week | 0.9967 | 2.71 (1.35, 5.42) | 0.0049 |
| Base-2 logarithm of adjusted cadmium concentration (ng/g)^b^ | 0.1748 | 1.19 (1.01, 1.41) | 0.0388 |
| CI = confidence interval  ^a^Body mass index is weight in kilograms divided by (height in meters)^2^  ^b^Adjusted by urine concentration of creatinine (mg/dL) | | | |
