# Supplementary material for: Cadmium exposure and endometrial cancer risk: A large midwestern U.S. population-based case-control study
Source: PLoS One. 2017 Jul 24;12(7):e0179360. doi: 10.1371/journal.pone.0179360 (PMC5524364; doi:10.1371/journal.pone.0179360)
Supplement: S4 Table — (DOCX) [file pone.0179360.s004.docx]

| S4 Table. Multivariable conditional logistic regression of risk factors for endometrial cancer, including age at menarche and number of live births. | | | |
| --- | --- | --- | --- |
| Characteristic | Parameter estimate | Odds ratio (95% CI) | P-value |
| Non-Hispanic African-American race | 1.6477 | 5.19 (1.96, 13.8) | 0.0009 |
| Marital status (reference never married) |  |  |  |
| Married, living with partner | -0.8466 | 0.43 (0.19, 0.97) | 0.0415 |
| Divorced, separated, widowed | -0.6269 | 0.53 (0.23, 1.26) | 0.1527 |
| Body mass index at diagnosis (5kg.m^2^)^a^ | 0.0822 | 1.51 (1.36, 1.67) | <0.0001 |
| History of trying to lose weight | 0.4693 | 1.60 (0.99, 2.58) | 0.0543 |
| Current smoker | -0.6764 | 0.51 (0.27, 0.94) | 0.0323 |
| Cigarette smoking (10 pack-years) | -0.0127 | 0.88 (0.80, 0.97) | 0.0091 |
| History of endometriosis | 0.4894 | 1.63 (1.08, 2.46) | 0.0199 |
| History of breast cancer | -0.9582 | 0.38 (0.16, 0.93) | 0.0329 |
| History of ovarian cancer | 2.3265 | 10.2 (2.73, 38.4) | 0.0006 |
| History of uterine fibroids | -0.3435 | 0.71 (0.50, 1.00) | 0.0528 |
| Endometrial cancer in first degree relative | 1.2175 | 3.38 (1.41, 8.08) | 0.0062 |
| Oral contraceptive use (5 years) | -0.0272 | 0.87 (0.79, 0.97) | 0.0114 |
| Unopposed estrogen use (5 years) | -0.0804 | 0.67 (0.53, 0.84) | 0.0007 |
| Menopause at age 56 or later | 0.5368 | 1.71 (1.13, 2.58) | 0.0105 |
| Post-menopausal at diagnosis | -1.1233 | 0.33 (0.21, 0.52) | <0.0001 |
| Protein shake consumption, days/week | 0.1851 | 1.20 (1.04, 1.39) | 0.0115 |
| Whole milk consumption, ≥ 5 days/week | 0.9871 | 2.68 (1.33, 5.43) | 0.0061 |
| Base-2 logarithm of adjusted cadmium concentration (ng/g)^b^ | 0.2146 | 1.24 (1.05, 1.47) | 0.0127 |
| Age at menarche | 0.0045 | 1.00 (0.92, 1.10) | 0.9204 |
| Number of live births | -0.0826 | 0.92 (0.83, 1.03) | 0.1361 |
| CI = confidence interval  ^a^Body mass index is weight in kilograms divided by (height in meters)^2^  ^b^Adjusted by urine concentration of creatinine (mg/dL) | | | |
