# Supplementary material for: Cadmium exposure and endometrial cancer risk: A large midwestern U.S. population-based case-control study
Source: PLoS One. 2017 Jul 24;12(7):e0179360. doi: 10.1371/journal.pone.0179360 (PMC5524364; doi:10.1371/journal.pone.0179360)
Supplement: S5 Table — (DOCX) [file pone.0179360.s005.docx]

| S5 Table. Multivariable conditional logistic regression of risk factors for endometrial cancer, including unadjusted cadmium concentration and creatinine concentration. | | | |
| --- | --- | --- | --- |
| Characteristic | Parameter estimate | Odds ratio (95% CI) | P-value |
| Non-Hispanic African-American race | 1.5888 | 4.90 (1.86, 12.90) | 0.0013 |
| Marital status (reference never married) |  |  |  |
| Married, living with partner | -1.0228 | 0.36 (0.17, 0.77) | 0.0082 |
| Divorced, separated, widowed | -0.8136 | 0.44 (0.20, 0.99) | 0.0472 |
| Body mass index at diagnosis (5kg.m^2^)^a^ | 0.0831 | 1.52 (1.37, 1.68) | <0.0001 |
| History of trying to lose weight | 0.4759 | 1.61 (0.99, 2.61) | 0.054 |
| Current smoker | -0.6741 | 0.51 (0.27, 0.96) | 0.0364 |
| Cigarette smoking (10 pack-years) | -0.0133 | 0.88 (0.80, 0.96) | 0.0052 |
| History of endometriosis | 0.5179 | 1.68 (1.11, 2.53) | 0.0133 |
| History of breast cancer | -0.9523 | 0.39 (0.16, 0.93) | 0.033 |
| History of ovarian cancer | 2.3092 | 10.1 (2.69, 37.7) | 0.0006 |
| History of uterine fibroids | -0.3474 | 0.71 (0.50, 1.00) | 0.049 |
| Endometrial cancer in first degree relative | 1.2392 | 3.45 (1.40, 8.49) | 0.0069 |
| Oral contraceptive use (5 years) | -0.0253 | 0.88 (0.79, 0.98) | 0.0178 |
| Unopposed estrogen use (5 years) | -0.0800 | 0.67 (0.53, 0.84) | 0.0007 |
| Menopause at age 56 or later | 0.5255 | 1.69 (1.12, 2.55) | 0.0117 |
| Post-menopausal at diagnosis | -1.1187 | 0.33 (0.21, 0.52) | <0.0001 |
| Protein shake consumption, days/week | 0.1824 | 1.20 (1.04, 1.39) | 0.0143 |
| Whole milk consumption, ≥ 5 days/week | 0.9437 | 2.57 (1.29, 5.13) | 0.0074 |
| Base-2 logarithm of unadjusted cadmium concentration (ng/g)^b^ | 0.2344 | 1.26 (1.07, 1.49) | 0.0051 |
| Creatinine concentration (mg/dL) | -0.0031 | 1.00 (0.99, 1.00) | 0.0552 |
| CI = confidence interval  ^a^Body mass index is weight in kilograms divided by (height in meters)^2^ | | | |
